# The relationship of social determinants and distress in newly diagnosed cancer patients

**DOI:** 10.1038/s41598-023-29375-5

**Published:** 2023-02-07

**Authors:** Brandon Okeke, Cheron Hillmon, Jasmine Jones, Grace Obanigba, Ann Obi, Meagan Nkansah, Nicholas Odiase, Kamil Khanipov, Ikenna C. Okereke

**Affiliations:** 1 School of Medicine, University of Texas Medical Branch, Galveston, TX USA; 2 Department of Care Management, University of Texas Medical Branch, Galveston, TX USA; 3 Department of Pharmacology and Toxicology, University of Texas Medical Branch, Galveston, TX USA; 4 Department of Surgery, Henry Ford Health System, 2799 W. Grand Blvd, Detroit, MI 48202 USA

**Keywords:** Cancer, Cancer epidemiology

## Abstract

Patients with a new cancer diagnosis can experience distress when diagnosed. There are disparities in treatment of cancer patients based on social determinants, but minimal research exists on the relationship of those social determinants and distress after a new cancer diagnosis. Our goals were to determine the social determinants associated with distress after a new cancer diagnosis and determine the relationship of distress with outcome. Patients with a new cancer diagnosis at one institution from January 2019 to December 2020 were analyzed. Patients were given the National Comprehensive Cancer Network (NCCN) distress thermometer during their first visit. Demographics, tumor characteristics, clinical variables and survival were recorded. Patients were also asked to share specific factors that led to distress, including: (1) financial, (2) transportation, (3) childcare and (4) religious. A total of 916 patients returned distress thermometers. Mean age was 59.1 years. Females comprised 71.3 (653/916) percent of the cohort. On Dunn’s multiple comparison, the following factors were associated with increased distress level: female (p < 0.01), ages 27 to 45 (p < 0.01), uninsured (p < 0.01) and unemployed (p < 0.01). Patients with higher distress scores also experienced worse overall survival (p < 0.05). Females, young patients, uninsured patients and unemployed patients experience more distress after a new cancer diagnosis. Increased distress is independently associated with worse overall survival. Social determinants can be used to predict which patients may require focused interventions to reduce distress after a new cancer diagnosis.

## Introduction

Patients who receive a new diagnosis of cancer experience various levels of distress. According to the National Comprehensive Cancer Network (NCCN) distress management guidelines, distress is characterized as an unpleasant mental, physical, social, or spiritual experience^1^. Distress can impact one’s thoughts, actions, and emotions, making it more difficult to cope with the cancer diagnosis, symptoms, and treatment^2^. Alterations in distress levels are often related to the anticipated modifications in a patient’s lifestyle, concerns about mortality, and lack of information regarding prognosis following the diagnosis. Additionally, it is believed that other preexisting factors, such as the level of educational attainment, marital status, or prior cancer diagnosis, also influence the magnitude of distress experienced^3^.

There is growing evidence that distress is highly prevalent yet under-recognized in newly diagnosed cancer patients^4,5^. High distress levels influence patients’ abilities to complete cancer treatments, alter their quality of life during and after cancer therapy and impact their overall health outcomes^6,7^. Therefore, it is critical to assess these levels before treatment begins. Additionally, disparities in treatment may lead to the level of distress experienced by patients. African Americans tend to be diagnosed at advanced stages compared to other races and have greater obstacles to obtain appropriate treatment^8–10^. African Americans have worse survival for most cancers in the United States^11^. African American and Hispanic women also fail to receive definitive local therapy for curable breast cancers more often than Caucasians after adjustment for age and tumor characteristics^12,13^. All of these factors can lead to increased distress in patients with a cancer diagnosis.

Social determinants of health are defined as the non-medical factors that influence health outcomes. These determinants can include race, gender, socioeconomic status, education level, housing and multiple other patient factors^14^. Social determinants of health can impact illness in multiple ways. Poverty can lessen access to medical caregivers^15^. Patients within lower socioeconomic statuses may have more medical mistrust and less health literacy^16,17^. Social determinants are also associated with negative modifiable behaviors such as smoking^18^.

**Figure 1:**
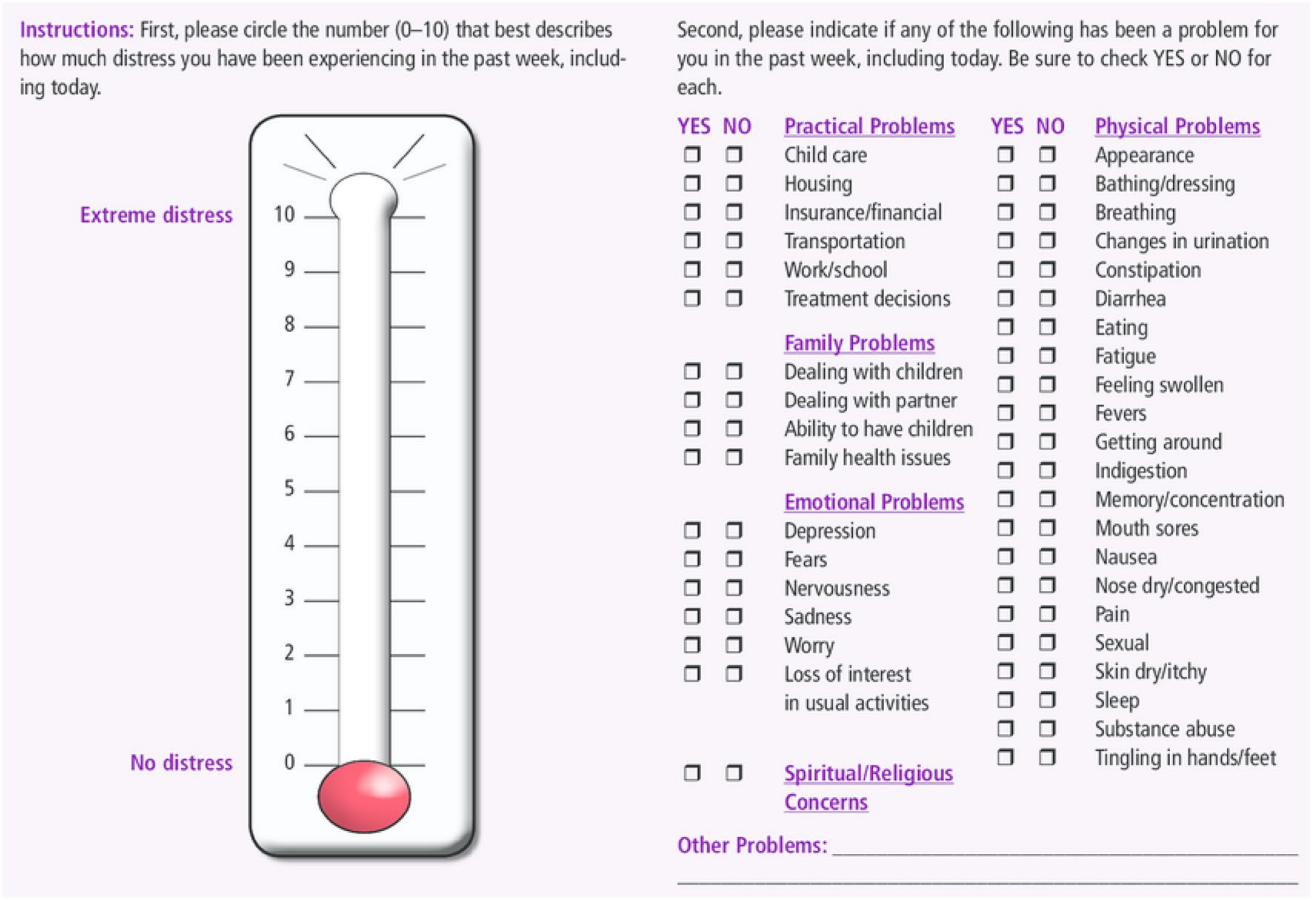
NCCN distress thermometer.

The most commonly used scale to measure distress is the NCCN distress thermometer, which ranges from 0 to 10 (Fig. 1). A rating of 0 implies no distress while a score of 10 implies extreme distress. The primary objectives of this study were to determine the risk factors associated with increased distress after a diagnosis of cancer, to determine prevalence of specific subsets of distress and to determine associations between increased distress and survival.

## Methods

### Patients

Institutional review board approval was obtained for the study and a waiver for consent was approved (IRB # 19-0289). All patients with a new diagnosis of cancer at our institution who completed an NCCN distress thermometer (Fig. 1) were included in our study. This study was a cross-sectional observational study across the institution. The study ranged from January 1st, 2019 to December 31st, 2020. All patients completed the distress thermometer during their first visit to our institution, immediately prior to seeing their physician. Patients who recorded a score of 6 or more were automatically referred to a social worker with their permission for specific assistance. This pattern of referral for scores of 6 or more were per institutional protocol. Once engaged with a social worker, patients were asked to share specific topics that may have led to the distress they were currently experiencing. These components were ultimately categorized as related to (1) financial, (2) transportation, (3) childcare, (4) religious or (5) other. After consultation, patients were offered available services to mitigate these concerns.

### Variables

The following data were collected for all patients: age, gender, race, distress thermometer score, insurance status, employment status, marital status, weight loss in last 6 months, smoking history, cancer type/location, stage, TNM status and survival status. All patients who had a distress score of 6 additionally were asked to answer yes or no for the presence of distress related to financial concerns, transportation concerns, childcare concerns, religious concerns or other concerns.

### Statistical analysis

To identify the appropriate statistical testing methods, all numeric variables were assessed for Gaussian distribution using Shapiro’s test, D’Agustino’s K2 test and the Anderson–Darling test. Statistical correlation analyses were performed to identify the correlation between distress scores and all other variables. Pearson, Kendall, and Spearman correlations were used for numeric data. Cramer’s V matrix and Theil’s U matrix were built for all variables as well. For the distress score and distress factors, Kruskal–Wallis one-way analysis of variance was performed. The Kruskal–Wallis test identified whether the samples originated from the same distribution. For each variable shown to be statistically significant, a post hoc analysis was performed. Dunn’s multiple comparison test allowed for detailed analysis of particular groups to determine statistically significant differences. A simple linear regression was conducted on the association between lower distress scores and overall patient survival. The regression was built based on the survival of patients who had a distress score between 1 and 9. Patients who scored their distress at either extreme (0 or 10) were excluded, as a way to minimize the bias due to extreme response styles^19^.

Additionally, using “Distress score” as the dependent variable and various features as independent variables, we conducted a series of statistical tests. An appropriate statistical test was chosen based on the type of independent variable—numerical, ordinal, or categorical. Categorical features were shown to be independent of each other using Cramer’s V test.

For the features with two categories (e.g., “Alive,” “Active smoker,” and “Referral to social work”), we conducted the standard two-tailed *t* test. All the tested features were shown to be statistically different in features with two categories. Note that “Alive” is considered a feature only in the statistical, not causal sense. Being alive was not modulated to affect the “Distress score.”

For the features with three or more categories (e.g., “Type of insurance,” “Employment,” and “Marital status”), F test for univariate ANOVA was conducted. The null hypothesis was rejected when the variability between groups (e.g., type of insurance) was more than a threshold multiple of the variability within groups. Significance indicated that there was a significant difference between groups.

For the features with numerical entries including “Age” and “Overall stage,” Pearson’s r test was performed. For the specific case of “Age,” since we binned the age groups in 9-year-intervals for visualization, we also conducted an F test for the binned data vs. the distress score, which also returned significant results. F-test was performed to test if the slope is significantly different from 0.

The differences in the distributions of patient factors was evaluated for patients who reported no distress (distress score = 0) with all other patients (distress score 1–10). Mann–Whitney test was used to compare the distributions of continuous factors (e.g., age). Chi-square with Yates’ correction was applied to categorical factors (e.g., sex).

### Ethical standards

All methods were performed in accordance with the relevant guidelines and regulations and was approved by an appropriate ethics committee.

## Results

### Patients demographics

A total of 916 patients were included in the study. Table 1 displays patient demographics. Mean age was 59.1 years (18–93 years). Females comprised 71 percent (653/916) of the cohort. The most common race was Caucasian (63%, 581/916), followed by African American (20%, 185/916), and Hispanic (14%, 126/916). Breast cancer was the most common cancer type (44%, 401/916), followed by gynecologic malignancies (15%, 138/916) and gastrointestinal malignances (11%, 97/916). Thirty-four percent of patients (315/916) experienced weight loss within the previous 6 months. Six percent (50/916) of patients were uninsured at the time of their diagnosis.

**Table 1:** Patient demographics.

Age, years (mean)	59.1
Gender	
Female	71.3% (653/916)
Male	28.7% (263/916)
Race	
Caucasian	63.4% (581/916)
African American	20.2% (185/916)
Hispanic	13.8% (126/916)
Other	2.6% (24/916)
Marital status	
Married	47.8% (438/916)
Other	52.2% (478/916)
Insurance	
Private	45.4% (416/916)
Medicare	36.0% (330/916)
Medicaid	13.1% (120/916)
Uninsured	5.5% (50/916)
ECOG	
0–1	84.9% (769/916)
2–4	15.9% (147/916)
Cancer location	
Breast	43.8% (401/916)
Gynecologic	15.1% (138/916)
Gastrointestinal	10.6% (97/916)
Urologic	9.8% (90/916)
Lung	8.6% (79/916)
Ear, nose, or throat	7.0% (64/916)
Other	5.1% (47/916)
Stage	
In situ/I/II	48.8% (447/916)
III/IV	51.2% (459/916)

### Distress scores

**Figure 2:**
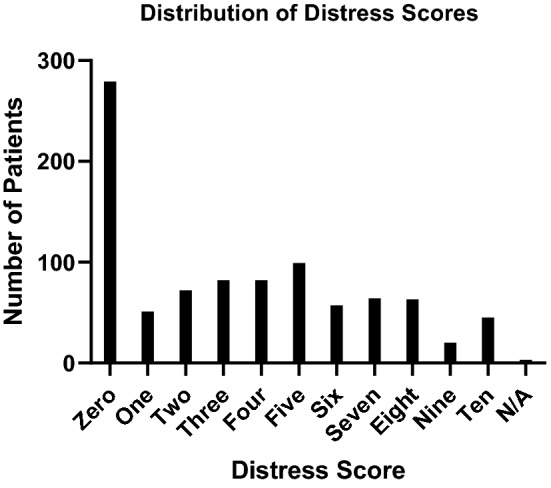
Distribution of distress scores.

Figure 2 shows the overall distribution of distress scores. The most frequent score was 0, in 31 percent (279/914) of patients. A total of 27 percent (249/914) of patients reported a distress score of 6 or greater, triggering an automatic referral to a social worker with the patient’s permission per institutional protocol.

### Factors associated with increased distress

**Figure 3:**
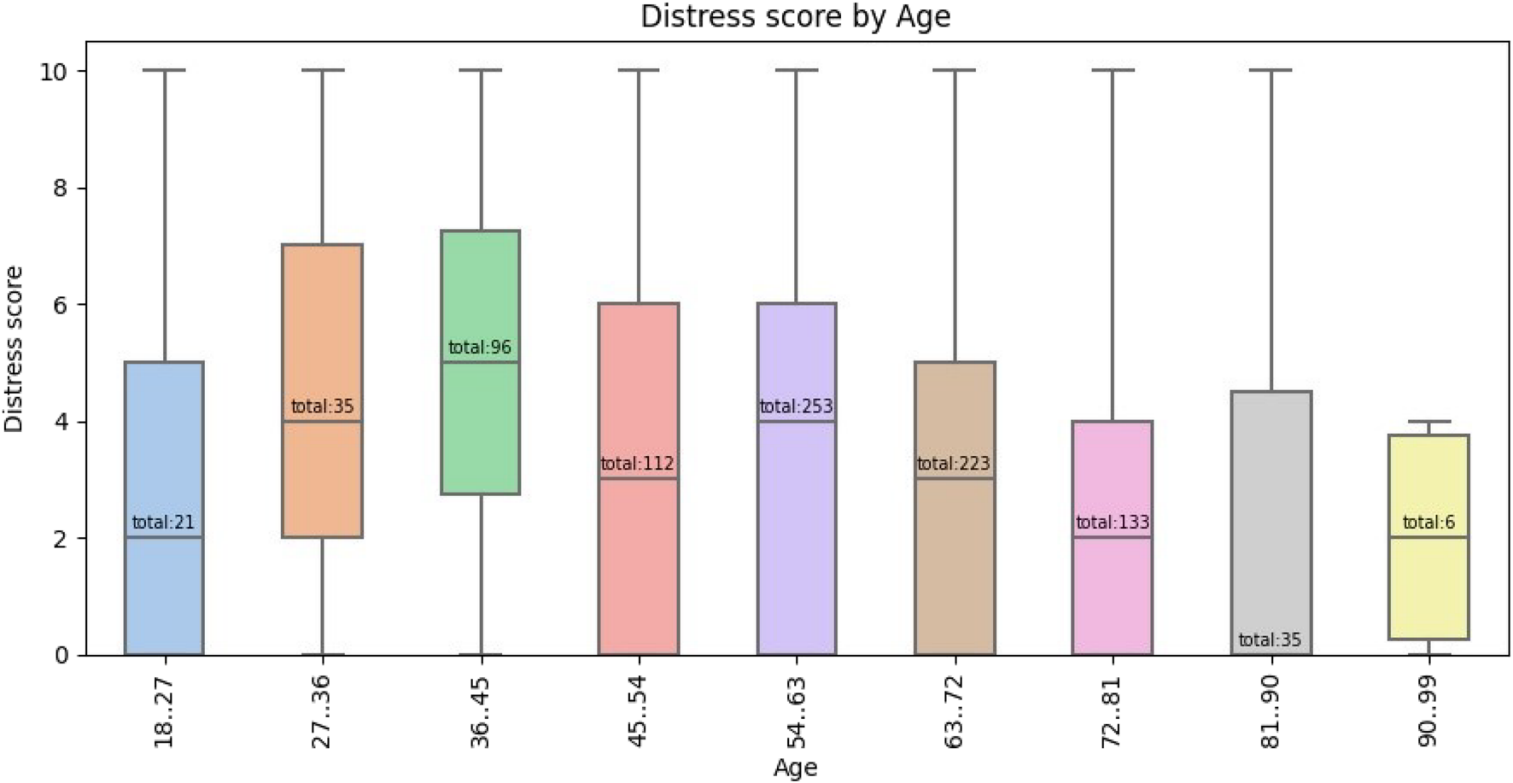
Distress scores by age.

Table 2 shows patient factors that are significant accord to Kruskal–Wallis test. Factors identified as significant on based on the Kruskal–Wallis test analysis were tested using Dunn’s multiple comparisons. The following factors persisted in being significantly associated with distress: female gender, ages 27–45, uninsured and unemployed. Figure 3 shows distress scores by age, with the highest values for ages 27–45.

**Table 2:** Factors significantly associated with increased distress on multivariate analysis.

Patient factor	p-value
Gender	< 0.001
Age	< 0.001
Type of insurance	< 0.01
Employment status	< 0.001
Active smoker	< 0.01
Cancer grouping	< 0.05
N stage	< 0.05
Referral to social work	< 0.001

### Specific distress components

Two hundred forty-nine patients were identified to have a distress score of 6 or greater. These patients were asked about specific components that were causing distress in their lives. The most common components which were causing distress in the referred group were financial struggles in 41% (103/249) of patients, transportation difficulties in 18% (44/249) of patients, religious conflicts in 17% (42/249) of patients and need for childcare in 5% (12/249) of patients.

### Distress score of 0

Given the large number of patients with a distress score of zero, this subgroup was compared to the rest of the cohort. Table 3 shows patient characteristics of each subgroup. Patients who gave a distress score of 0 were more likely to be younger, male and non-Caucasian.

**Table 3:** Patient characteristics of distress score of 0 versus all other distress scores. Significant values are in bold.

	Distress score = 0	Distress score 1–10	p-value
N	279	635	
Age (mean)	60.7 ± 14.7 years	58.4 ± 13.7 years	**0.02**
Female	62.0% (173/279)	75.4% (479/635)	**< 0.0001**
Caucasian	54.5% (152/279)	67.4% (428/635)	**< 0.001**
Married	47.7% (133/279)	48.9% (305/635)	0.98
Insured	94.3% (263/279)	94.7% (601/635)	0.94
Stage I/II	56.6% (158/279)	59.2% (376/635)	0.51
Alive	92.5% (258/279)	92.8% (589/635)	0.99

### Race/ethnicity

Race/ethnicity was not found to be significant in higher distress scores when including all patients (ANOVA F-test p = 0.26) or excluding patients who reported a distress score of zero (ANOVA F-test p = 0.54).

### Survival

**Figure 4:**
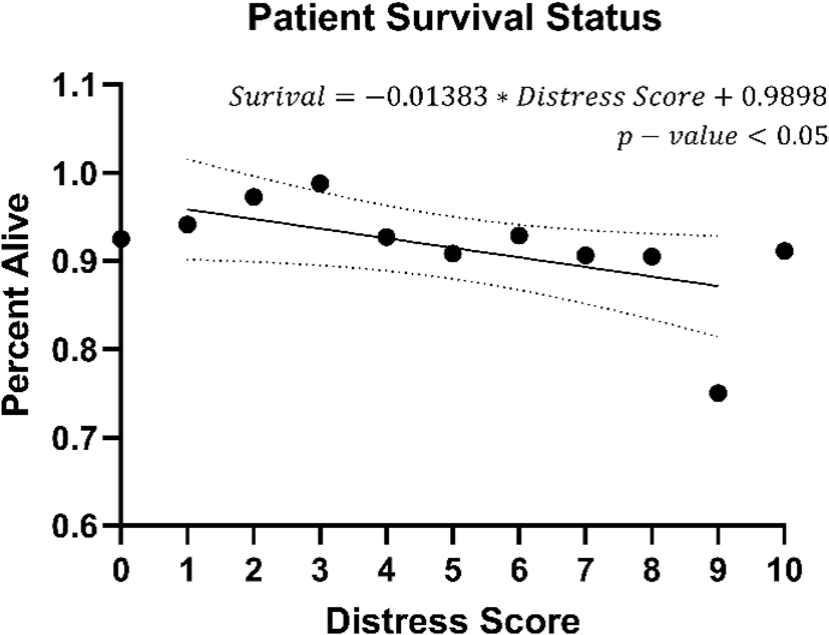
Survival status of patients per distress score.

Figure 4 shows survival of patients with a score of 1–9. As the distress score increased, patients were less likely to be alive at the end of the 2-year study period. Specifically, each additional point on the distress score was associated with a 1.38% decrease in overall survival. The slope was shown to be significantly nonzero (p = 0.04).

## Discussion

Our study revealed certain demographic variables that correlated with increased distress in patients with a new diagnosis of cancer. In particular, patients who were uninsured were more likely to have an increased distress score. Multiple studies have shown that lack of insurance is associated with worse outcomes for multiple disease processes^20–22^. Although lack of insurance is associated with numerous social determinants of health, our studies showed that not having insurance was independently associated with increased distress. As the uninsured rate has increased in our country in recent years^23^, more programs should be created which actively engage the uninsured population^24^. Creating cancer screening programs to target these communities will help to identify their cancers earlier and improve accessibility to health care centers for the uninsured population.

We chose to use the distress thermometer because it was a basic and very easy-to-use survey for patients. But we acknowledge that this thermometer lacks specific detail and is very general. The NCCN developed this distress thermometer in 1999 as a simple tool to screen patients quickly^25^. Over the last 20 years, however, the medical profession has developed much more sophisticated tools to measure distress. While we feel that this specific thermometer was useful as a general tool, we feel that more information and actionable processes will be identified with newer, more detailed surveys.

Most importantly, our studies showed that patients who have an increased distress score at presentation were more likely to have died during the study period. Our study in combination with other studies strongly suggests that some measurement of distress should be performed on every patient during the initial visit^26,27^. In addition, there is likely some utility in recording some measurement of this stress during each patient visit.

Our study also showed that women were more likely to improve experience increased distress levels with a new cancer diagnosis. Women may be more likely to have a broad set of responsibilities at home and in their careers^28^. Understanding this broad set of responsibilities can help healthcare institutions to provide avenues to decrease the burden that women face. Healthcare institutions may be able to reduce overwhelming feelings that young women face with areas for childcare during treatment, counseling available for entire families and support groups. These types of assistances are likely to help to decrease distress levels. Previous literature has shown that elevated levels of depression in women with a cancer diagnosis was associated with worse survival. When the level of depression was adjusted for anxiety levels, in fact, the strength of the association between depression and survival increased in women^29^.

In our study, young patients were more likely to have increased distress. The active lifestyles of younger patients and the unexpected nature of a cancer diagnosis in this age group may explain these increased levels of distress^30^. In our study, it was specifically patients between the ages of 27 and 45 with the highest distress scores. Having an appreciation that younger patients may be more susceptible to distress is important. When considering which patients require social work or counseling support, it may be beneficial to include a patient's age as one of the factors used in deciding who requires a referral. Our study is one of several which has shown this inverse association between age and distress in patients with a diagnosis of cancer^31,32^. Other studies used other survey instruments, and it appears that the NCCN distress thermometer was also able to yield similar findings.

We analyzed patients who reported a distress score of zero. We were interested in seeing if reporting a score of 0 was associated with any hesitancy in revealing distress. Interestingly, we showed that younger patients, minorities and men were more likely to give a score of zero. Although this may seem to contradict our previous finding that younger patients have more distress, we hypothesize that there may be a distress score hesitancy with some patients. Previous literature has shown that some people in society are less likely to seek care for illnesses. Future studies are needed, but there may be some utility in scrutinizing patients who report a distress score of 0 and measuring particular risk factors and outcomes in these patients. Our study was not large enough to examine these associations in greater detail.

When we examined specific factors leading to distress in patients with a score of six or greater, psychiatric and financial concerns were the most common reasons. Numerous studies have shown that impoverished patients have less accessibility to healthcare and worse outcomes with many diseases^33,34^. When a new patient presents to an institution, there are many sociodemographic variables that are recorded. Although components of social economic status are not recorded per se, there may be some utility in addressing a patient's financial health when they present with a new cancer diagnosis. There has to be an appreciation for patient confidentiality and respect for patients, but there may be some general questions that can be used to screen for vulnerability to distress based on financial health.

There are limitations to our study. Although our study analyzed almost 1000 patients, this was a fraction of the patients who presented with a new diagnosis of cancer during the time period. There was likely a selection bias in who decided to return a survey. But we could not make this survey obligatory, and we do feel that there was a relatively large number of patients which does add some value to the study results. Although the study included a wide number of cancer types, there may have been some bias in which patients decided to complete a distress survey, as this was optional. Other similar studies have faced this same dilemma, however, as mandating a survey in these circumstances can be problematic and add a burden to some patients who do not want to complete a survey. In addition, our study only obtained a distress score during the initial patient visit. Going forward, our institution will obtain a distress score during every patient visit. Finally, the secondary screening that we performed for elevated initial scores did not include important determinants of health. We will augment our secondary screening in the future.

Our study revealed important findings. It showed that there are certain social determinants that are associated with increased distress in patients with a new diagnosis of cancer. Furthermore, it showed that patients with an increased level of distress at diagnosis experience worse overall survival. Interventions in addressing social determinants and their relationship to distress will require focused action across practice, research, and policy domains.

## Data Availability

The data is available upon request. Please send an email request to iokerek1@hfhs.org.
